# Ultrasound Assessment of Synovial Thickness of Some of the Metacarpophalangeal Joints of Hand in Rheumatoid Arthritis Patients and the Normal Population

**DOI:** 10.1155/2016/5609132

**Published:** 2016-04-13

**Authors:** Zuhudha Hussain Manik, John George, Sargunan Sockalingam

**Affiliations:** ^1^University of Malaya Research Imaging Centre, Faculty of Medicine, University of Malaya, Kuala Lumpur, Malaysia; ^2^Department of Medicine, Faculty of Medicine, University of Malaya, Kuala Lumpur, Malaysia

## Abstract

*Objective*. To compare ultrasound synovial thickness of the 2nd, 3rd and 4th metacarpophalangeal joints (MCPJ) in a group of patients with proven rheumatoid arthritis (RA) and a control group of normal individuals.* Materials and Methods*. This is a cross-sectional study comprising 30 rheumatoid arthritis patients and 30 healthy individuals. Ultrasound scans were performed at the dorsal side of 2nd, 3rd, and 4th MCPJ of both hands in RA patients and the healthy individuals. Synovial thickness was measured according to quantitative method. The synovial thickness of RA patients and healthy individuals was compared and statistical cut-off was identified.* Results*. Maximum synovial thickness was most often detected at the radial side of the 2nd MCPJ and 3rd MCPJ and ulnar side of the 4th MCPJ of both hands which is significantly higher (*p* < 0.05) in RA patients compared to healthy individuals. With high specificity (96%) and sensitivity (90%) the optimum cut-off value to distinguish RA patients and healthy individuals' synovial thickness differs for the radial side of the 2nd and 3rd MCPJ and ulnar side of the 4th MCPJ.* Conclusion*. Patients with early RA appear to exhibit a characteristic pattern of synovitis which shows radial side predominance in the 2nd and 3rd MCPJ and ulnar side in the 4th MCPJ.

## 1. Introduction

Rheumatoid arthritis (RA) is a chronic systemic inflammatory disease that, if left untreated, will eventually cause progressive joint destruction and deformity resulting in irreversible long term disability [1].

RA has a favourable outcome if diagnosed early and treated aggressively if needed with disease-modifying antirheumatic drugs [2–4]. Synovitis of small joints of the hands is an early finding in RA. Untreated synovitis is known to be associated with irreversible damage in the joints, tendons, and ligaments [5]. Therefore, the early recognition of reversible synovitis in RA and close monitoring of disease activity are of great importance to avoid the likelihood of persistent disease and irreversible joint damage.

There are no universally agreed absolute measurements of synovial thickness in normal metacarpophalangeal joints (MCPJ). The proposed values are largely variable and different workers have used different joints and anatomical structures to define normal sizes [6–8]. Some studies showed that when scoring the synovial lining in normal subjects using grey-scale ultrasound (US) scoring systems for synovial hypertrophy, high number of joints was scored as pathological. It also showed that grade 1 of semiquantitative scoring was not specific for RA patients but was detected in the joints of healthy individuals [9–11]. This indicates that grey-scale ultrasound scores interpreted as pathological in patients with RA may sometimes be normal findings. Hence it is important to define standard reference values for normal synovial thickening in healthy individuals to prevent misinterpretation of synovial thickening as pathological.

Current recommended approach is to scan the MCPJ and proximal interphalangeal joints (PIPJ) of the hand in a midline sagittal approach for MCPJ and PIPJ scan using ultrasound [12]. Most of the authors used the volar aspect of the palm for scanning MCPJ and recommended scanning of the volar side [6, 8]. None of these previous ultrasound examinations mentioned whether they included the radial or ulnar side of MCPJ to assess synovitis, even though there is predilection of maximum synovitis in the radial and ulnar side of MCPJ [13, 14]. Also although previous studies recommended dorsal or volar synovial measurement of MCPJ [6, 8, 12], they have not mentioned where exactly to measure the synovial thickness such as dorsal radial, dorsal midline, or dorsal ulnar sides. Hence our main aims were to assess distribution of maximum pathological synovitis of 2nd to 4th MCPJ using a standard bony landmark as well as site and quantify the maximal thickness of synovium in a control group and those with proven RA patients and then statistically assess its significance.

## 2. Methods and Materials

In this prospective study we recruited 30 RA patients, fulfilling the 2010 American College of Rheumatology/European League Against Rheumatism (2010 ACR/EULAR) classification criteria for RA [15] with disease duration of not more than 12 months and 30 healthy volunteers whose age and sex matched (±2 years) to RA patients. The inclusion criteria for healthy controls were those that had negative rheumatoid factor, anticyclic citrullinated peptide antibody, and C-reactive protein, with no joint pain or known systemic diseases that cause arthritis. The control patients also had no clinical manifestations or systemic features of arthritis or synovitis and no previous surgery or trauma to the joints examined. For healthy controls blood samples for C-reactive protein, rheumatoid factor and anticyclic citrullinated peptide antibody were taken to ensure they were within normal limits. RA patients were selected from the Rheumatology Clinic at our Center. Staffs and their relatives at our Center were recruited as healthy controls on an age and sex matched basis. The study was performed with the approval of the Medical Ethics Committee of University of Malaya Medical Centre (reference number 872.23). All patients and healthy controls gave informed consent to participate in the study.

### 2.1. Ultrasound Examination

Ultrasound examination of MCPJ was performed using a Philips IU22 ultrasound machine (Philips Healthcare, Best, Netherlands) with multilinear 15-7io MHz hockey stick transducer. All examinations were performed by two investigators, a musculoskeletal radiologist with 18 years of experience in musculoskeletal radiology and a senior radiology trainee. The investigators measured synovial thickness independently.

**Figure 1:**
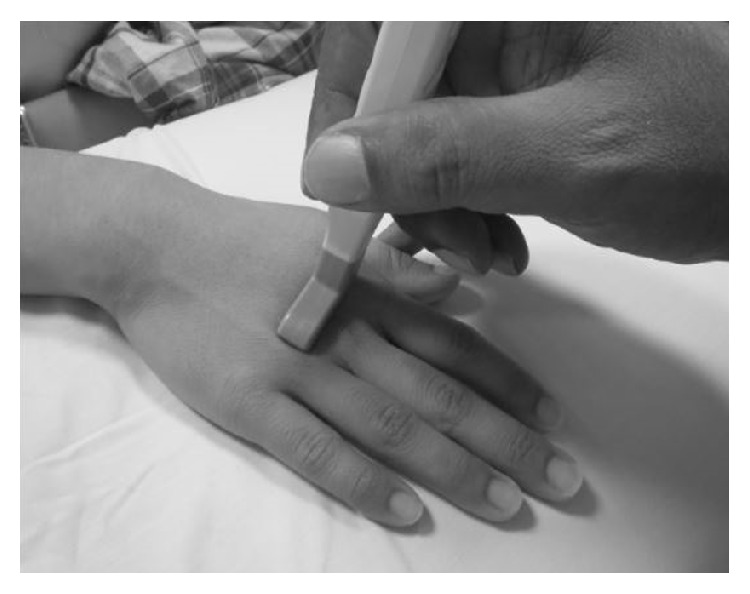
Position for examination of the hands. The neutral dorsal transverse position of the transducer in line with the radial and ulnar bare area of the metacarpophalangeal joint.

**Figure 2:**
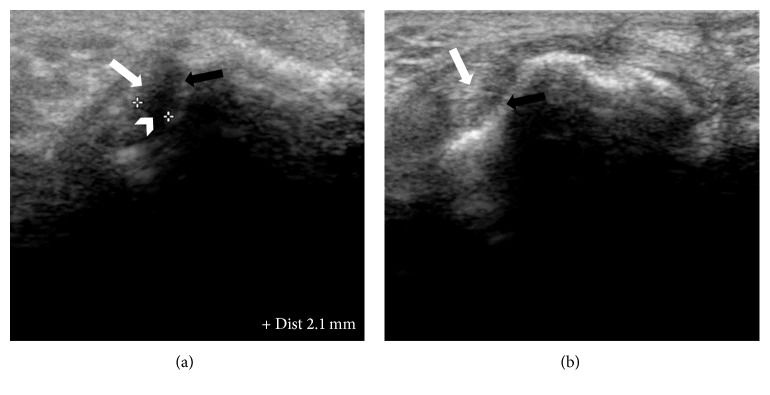
Dorsal ultrasound images of MCPJ at bare areas. (a) Radial recess shows distinct hypoechoic synovium (*arrow head*) compared to displaced hyperechoic proper collateral ligament (*white arrow*) and bone (*black arrow*). Calipers are being used to measure the maximal synovial thickness at this region with characteristic appearance on ultrasound. (b) shows no synovial thickening between the proper collateral ligament (white arrow) and adjacent to cortical bone (*black arrow*).

From a pilot study that was conducted at University Malaya Medical Center, the musculoskeletal radiologist involved in this study had observed that the maximal synovitis in early RA patients was found at the radial side of the 2nd and 3rd MCPJ (bare area) and the ulnar side of the 4th MCPJ (bare area), the latter not being described in the literature. The difference in the thickness between the radial and ulna sides appeared to have statistical significance which deserved a more formal study. The bare area of a small joint is the periarticular noncartilage region along with the small joint capsule. This finding of maximal synovitis at the radial sides of the 2nd, 3rd, and ulnar side of the 4th MCPJ appears to be supported by magnetic resonance imaging (MRI) studies [14]. Therefore in our study a transverse scan across the bare area of the 2nd to 4th MCPJ within the synovial capsule was standardised as the scanning plane and used to take the measurements of maximal synovitis of the bare areas of radial and ulna sides of these joints. The hockey stick probe is placed transversely initially over the extensor surface of the MCP joint (Figure 1) covering both radial and ulnar bare area where the bare areas show a slight concavity in bony contour and then the probe is moved slightly in the radial direction and ulna direction to make both the proper collateral ligament and adjacent bony cortex of the bare area appear hyperechoic and eliminate anisotropy (Figure 2). Measurement is then taken of the hypoechoic area from the bare area cortex to the proper collateral ligament (Figure 2) which should be only due to synovial thickening at the bare area and not synovial fluid as only synovitis with mass effect can displace the proper collateral ligament. Synovial fluid should be displaced into the volar recess of the joint which coincidently is one of the regions used for measurements of synovitis in conventional methods. Thus the strengths of this technique is a reproducible bony landmark, with ease of identifying and measuring hypoechoic synovitis and elimination of hypoechoic joint fluid being included in the measurements.

The characteristic bony landmark (shape) of the metacarpal head at which measurement of the bare area is made of the radial and ulnar side synovitis under the proper collateral ligaments allowing reproducible measurements to be made. This would allow for follow-up scans to assess effectiveness of the targeted management on the thickness of synovitis as the measurements of the bony width of the dorsal aspect of the metacarpal head at the level of the bare area can be used to ensure consistency of the point at which the radial and ulnar thickness of synovitis is measured. The dorsal side of the 2nd, 3rd, and 4th MCPJ of both hands was examined and measured according to Outcome Measures in Rheumatology Clinical Trials (OMERACT) consensus [16]. Grey-scale synovitis was graded using a quantitative scoring method as per McNally's article [12] as below; grade 0: <0.5 mm, grade 1: 0.5–2 mm thickness, grade 2: 2–4 mm thickness, and grade 3: >4 mm thickness. For healthy controls also the same ultrasound parameters were assessed.

### 2.2. Statistical Analysis

Statistical analysis was performed using SPSS (Statistical Package for Social Science) version 17 software. Statistical tests performed included the Mann-Whitney *U* tests and Wilcoxon Signed-Rank test to analyse difference between means of nonparametric data. For the demographic data, simple descriptive statistics were performed. The confidence interval for test significance was set at 95% with a significant *p* value of 0.05 or less.

To confirm ability of US to discriminate between patients with early RA and healthy subjects an analysis of receiver-operating characteristic (ROC) curves was performed.

## 3. Results

### 3.1. Descriptive Study

A total of 30 RA patients were recruited in this study. The patients consist of 63.3% (*n* = 19) seropositive RA and 36.7% (*n* = 11) seronegative RA. Patients were aged between 21 and 70 years, with a mean age of 47.37 ± 14.17 years, out of which 22 (73.3%) patients were female and 8 (26.7%) patients were male.

The control group consists of 30 healthy individuals. The mean age of this group was 46.63 ± 14.30 years. There were 70% of females and 30% of males in this group.

### 3.2. Synovitis Score

#### 3.2.1. Synovitis Grading in Rheumatoid Arthritis Patients

**Figure 3:**
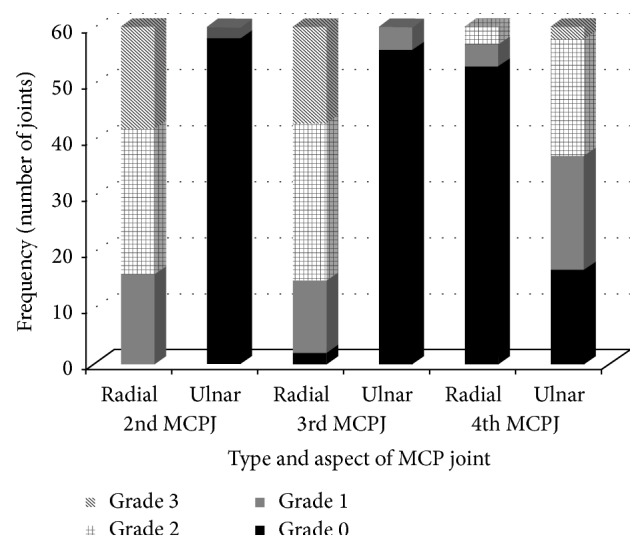
Distribution of grades of synovitis in the radial and ulnar sides of the 2nd, 3rd and 4th MCPJ of both hands in RA patients.

A total of 180 joints of RA patients were studied to assess synovial thickness in B mode ultrasound (2nd, 3rd, and 4th MCPJ of both hands).  Figure 3 shows the various grades of synovitis detected at the 2nd, 3rd, and 4th MCPJ at the radial and ulnar sides. It was found that in the 2nd and 3rd MCPJ, the predominantly involved side is the radial side. In these two joints the ulnar side synovitis appeared significantly less than the radial side. In the 2nd and 3rd MCPJ the predominant grade of synovitis was of grade 2 followed by grade 3. In contrast synovitis of the 4th MCPJ predominantly involved side was the ulnar side; the radial side of the 4th MCPJ was rarely involved. In the 4th MCPJ the frequency of grade 2 and grade 3 synovitis was less as compared to the 2nd and 3rd MCPJ.

#### 3.2.2. Synovitis Grading in Healthy Control Group

**Figure 4:**
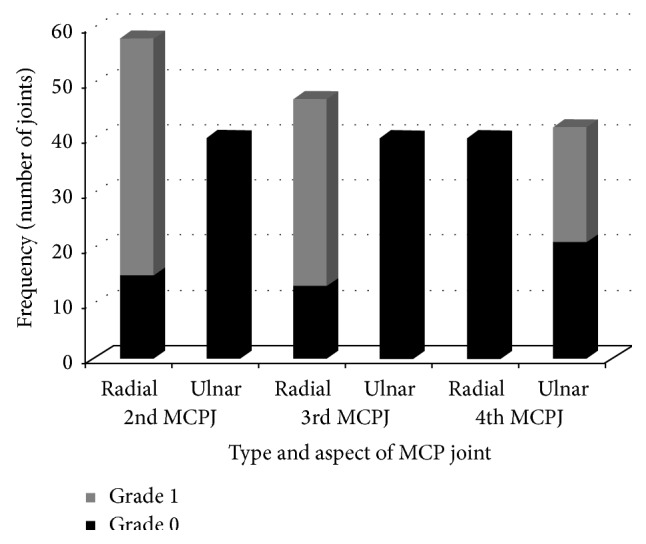
Distribution of grades of synovitis in the radial and ulnar sides of the 2nd, 3rd, and 4th MCPJ of both hands in the healthy control group.

A total of 180 joints of healthy control group were studied to assess synovial thickness in B mode ultrasound (2nd, 3rd, and 4th MCPJ of both hands); Figure 4 shows grades of synovitis distribution in healthy control group.

There was grade 1 synovitis in 98 joints (54.4%). In this control group there was no grade 2 or grade 3 synovitis. Grade 1 synovitis was seen in 43.8% of subjects in the radial side of the 2nd MCPJ of both hands. Grade 1 synovitis was also seen in 34.7% subjects in the radial aspect of the 3rd MCPJ. Ulnar side of the 2nd and 3rd MCPJ was normal in all the subjects. Radial side of the 4th MCPJ was normal in all the subjects whereas grade 1 synovitis was seen in 21.4% of subjects in the ulnar side of the 4th MCPJ.

#### 3.2.3. Synovitis Distribution

**Figure 5:**
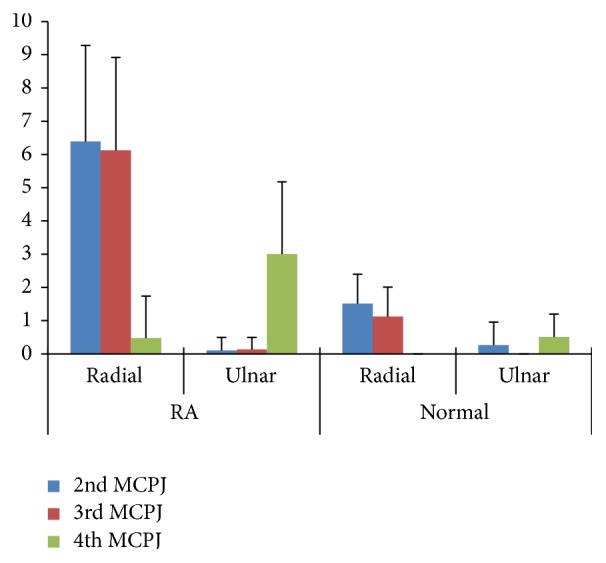
Box plot presentation of synovial thickness distribution of 2nd, 3rd, and 4th MCPJ in RA patients and healthy control group.

In both hands, the mean synovial thickness was highest in the radial side of the 2nd MCPJ followed by the radial side of the 3rd MCPJ. However, the 4th MCPJ in both the hands showed a greater mean synovial thickness on the ulnar side as compared to the radial side. There was significantly more synovitis at the radial side of the 2nd and 3rd MCPJ and ulnar side of the 4th MCPJ. (*p* < 0.05; Wilcoxon Signed-ranked test).  Figure 5 shows the distribution of synovitis in RA patients and healthy control group.

When synovial thickness of RA patients was compared with the control group, there was significantly higher synovitis at the radial side of the 2nd and 3rd MCPJ and ulnar side of the 4th MCPJ of rheumatoid arthritis patients compared to healthy control group (*p* < 0.05; Mann-Whitney *U* test). Meanwhile there was no significant difference in synovial thickness of ulnar side of the 2nd and 3rd MCPJ and radial side of the 4th MCPJ of RA patients and control group.

We did not find significant difference in distribution of synovitis in any of the MCPJ of seropositive RA patients compared to seronegative RA patients (*p* > 0.05).

**Figure 6:**
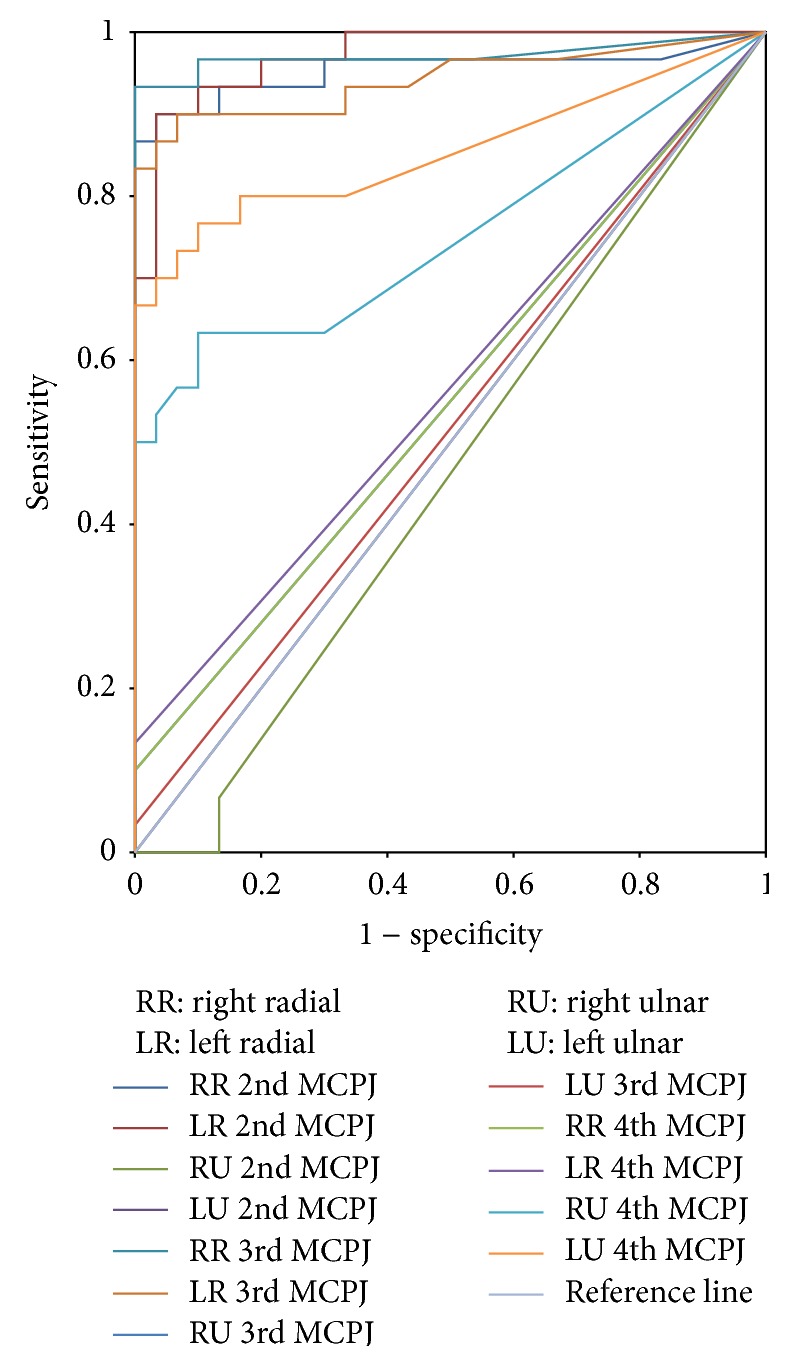
ROC curve.

To confirm the ability of ultrasound to discriminate between patients with early rheumatoid arthritis and healthy subjects, ROC curves were performed (Figure 6). It showed good discrimination of synovial thickness of the rheumatoid joints and healthy joints. The asymptotic significance test was statistically significant (*p* value < 0.05) for the radial side of the 2nd MCPJ, radial side of the 3rd MCPJ, and ulnar side of the 4th MCPJ. With high specificity and sensitivity the optimum cut-off value to distinguish RA patients and healthy individuals' synovial thickness varies in each of these MCPJ as shown in Table 1. The area under the curve was highest for the radial side of the left 2nd MCP joint (0.972, *p* < 0.001). Hence, this would be the best single joint to assess the synovial thickness to differentiate between healthy and diseased joints.

Ulnar side synovial thickness of the 2nd and 3rd MCPJ is uninformative in discriminating RA patients from healthy controls as the area under the ROC curve is not significantly different from 0.5 (*p* > 0.05). Similar findings were observed at the radial side synovial thickness of the 4th MCPJ.

### 3.3. Interobserver Agreement

The two investigators who measured the synovial thickness independently and were blinded to each other's findings got a highly reliable scoring as shown by the intraclass correlation coefficient (ICC) statistics of 0.944 (with a 95% confidence interval of 0.934-0.952).

## 4. Discussion

Synovitis is an important predictor of outcome in RA. The role of US in detecting synovitis in early RA and in predicting disease progression is well known [17–19]. However, it is difficult to establish a clear cut-off between healthy synovial thickness and synovitis in RA patients. Earlier studies suggested that there is potential for overdiagnosis of healthy individuals as pathological using current recommended scoring systems. Hence, our main aim is to assess synovial distribution in early RA patients and healthy controls.

We found most of the synovitis at the radial side of the 2nd and 3rd MCPJ and the ulnar side of the 4th MCPJ. The maximum synovitis at the radial side of 2nd and 3rd MCPJ is in concordance with previous study of Hau et al. and Tan et al. [13, 14]. However, Tan et al. found equal distribution of synovitis in both radial and ulnar sides of 4th MCPJ in contrast to our study. The maximal synovitis on the ulnar side of the 4th MCPJ in control and RA patients has not been described in the previous literature. Our findings disagree with the location of maximum synovitis in MCPJ, as compared to other studies. Scheel et al. found more synovitis in the palmar and proximal sites of MCPJ and PIP joints with only 14% joints showing synovitis on the dorsal side. Effusion and synovial hypertrophy were not separately considered but used as a combined measure which may provide inaccurate measurements than assessment of just synovial thickening which is the important measurement to distinguish between pathological and normal synovial thickness [6]. In 2011, Vlad et al. stated that volar synovitis is greater than dorsal synovitis and prevalence of synovitis is higher (88.1%) in volar side of 2nd MCPJ [8]. Ulnar and radial sides were not examined in these studies.

Although there is significantly greater synovial thickness in early RA patients compared to healthy control group we observed that the pattern of distribution of normal synovial thickness is similar to RA patients; that is, greater synovial thickness was noted on the radial side of 2nd and 3rd MCPJ and the ulnar side of 4th MCPJ.

In our study there is significant proportion of healthy individuals with US quantitative grade 1 synovial thickness, which was consistent with the results of a number of earlier studies. However in contrast to those studies we measured synovial thickness quantitatively. Millot et al. found 62% of healthy joints had grade 1 grey-scale semiquantitative synovitis and 11% of bone erosion [9]. Ellegaard et al. reported when scoring the synovial lining in normal subjects using grey-scale scoring systems that an unacceptably high number of joints were scored as pathological, with increasing numbers in older patients [11]. Witt et al.'s study also observed grade 1 synovial thickening in semiquantitative ultrasound scoring in healthy controls [10]. This increased finding of grade 1 synovial thickness in healthy individuals could be the fact that newer transducers and ultrasound machines are more sensitive in detecting small fluid in the joint.

To identify optimal threshold to distinguish early RA patients and healthy controls, ROC analysis was done. Previous studies used one measurement for all joints as the best cut-off value, unlike our study. We have measured synovial thickness for each of the 2nd, 3rd, and 4th MCPJ separately. It showed with good sensitivity and specificity the best cut-off value to distinguish early RA patients and healthy controls vary for different MCPJ (Table 1). For example, best cut-off value to distinguish early RA patients from healthy controls for right 2nd MCPJ and right 3rd MCPJ radial side was 1.7 mm and 1.3 mm, respectively. We also observed that when using the previous study cut-off value of 0.6 mm recommended by Scheel et al. [6], a large number of normal individuals could be regarded as pathologic, thus misdiagnosing a healthy individual as an RA patient. Schmidt et al. proposed mean value of 1.9 mm as normal which would label rheumatoid arthritis patients as normal individuals [7]. In this study we found a characteristic pattern of maximum synovitis, involving the radial side of 2nd and 3rd MCPJ and the ulnar side of the 4th MCPJ. Our results showed that combining maximum cut-off values with this characteristic synovitis distribution pattern may improve early diagnosis of RA patients and can avoid labelling normal individuals as RA and vice versa.

Ultrasound is a highly operator dependent modality which needs a reproducible method of measurement of synovial thickness with very good interobserver reliability. As in previous studies, good interobserver reliability of scan using dorsal transverse approach is proven from our study as a radiology trainee having very less musculoskeletal ultrasound experience achieved a good interobserver agreement with an experienced musculoskeletal specialist [20, 21]. As of now, there was no global agreed method of synovial thickness measurement [22]. Even though previous studies recommended dorsal or volar synovial measurement of MCPJ [6, 8, 12], they did not mention where exactly to measure the synovial thickness such as dorsal radial, dorsal midline, or dorsal ulnar sides. Therefore, we propose to measure synovial thickness at the radial and ulnar bare areas of the metacarpal head as described earlier where the distinct appearance of hypoechoic synovium appears, as it is the point where maximum synovitis is present in MCPJ.

## 5. Conclusion

In conclusion ultrasound measurement of synovial thickness is a valuable tool to distinguish early rheumatoid arthritis patients from healthy individuals. Patients with early RA have a characteristic distribution of synovial thickness with statistically significant maximal radial synovitis of the 2nd and 3rd MCPJ and ulnar synovitis of the 4th MCPJ compared to the normal individuals. This finding may be helpful in early diagnosis of seronegative RA or differentiate it from other common arthropathies. When transverse dorsal approach is utilised to scan MCPJ of the hands, the true maximum synovitis can be measured without including synovial fluid as only synovial mass can displace the proper collateral ligament and synovial fluid due to the tautness of the proper collateral ligament. Further studies with larger number of subjects and comparison with other types of arthropathies will be needed to validate these important findings which may assist physicians for early diagnosis of seronegative RA and differentiate it from other arthropathies.

## Figures and Tables

**Table 1:** Optimal cut-offs to distinguish between the synovial thickness of the healthy and pathological joints.

MCP joint	Synovial thickness (mm)	Sensitivity (%)	Specificity (%)
Radial right 2nd MCP joint	1.72	90	96.7
Radial left 2nd MCP joint	1.50	90	96.7
Radial right 3rd MCP joint	1.35	93	100
Radial left 3rd MCP joint	1.46	90	93.7
Ulnar right 4th MCP joint	1.06	53	96.7
Ulnar left 4th MCP joint	1.15	70	96.7
